# Association between infant birthweight and pelvic floor muscle strength: a population-based cohort study

**DOI:** 10.1186/s12884-023-05511-x

**Published:** 2023-04-19

**Authors:** Junyan Zhu, Junwen Si, Lu Zhao, Wei Liu

**Affiliations:** 1 Department of Gynecology and Obstetrics, Renji Hospital, School of Medicine, Shanghai Jiaotong University, Shanghai, 200127 China; 2 Department of Gynecology and Obstetrics, Jiuting Hospital, Shanghai, 201615 China

**Keywords:** Birthweight, Natural childbirth, Muscle strength, Pelvic floor, Pelvic floor disorders

## Abstract

**Background:**

To assess the relationship between infant birthweight and pelvic floor muscle (PFM) strength in China.

**Methods:**

We performed a retrospective, single-center cohort study of 1575 women delivering vaginally between January 2017 and May 2020. All participants completed pelvic floor examinations within 5–10 weeks after delivery and were evaluated for PFM strength, which was estimated by vaginal pressure. Data were collected from electronic records. We evaluated the association between infant birthweight and vaginal pressure through multivariable-adjusted linear regression analysis. We also performed subgroup analyses stratified by potential confounders.

**Results:**

Vaginal pressure decreased as the quartile of birthweight increased (*P* for trend < 0.001). Beta coefficients were -5.04 (95%CI -7.98 to -2.1), -5.53 (95%CI -8.5 to -2.57), -6.07 (95%CI -9.08 to -3.07) for birthweight quartile 2–4, respectively (*P* for trend < 0.001), independent of age, postpartum hemorrhage, and the number of vaginal deliveries. In addition, the results of subgroup analyses showed the same patterns across strata.

**Conclusions:**

This study demonstrates that infant birthweight was associated with decreased vaginal pressure in women after vaginal delivery and could be considered a risk factor for decreased PFM strength in the population with vaginal delivery. This association may provide an extra basis for appropriate fetal weight control during pregnancy, and for earlier pelvic floor rehabilitation of postpartum women delivering babies with larger birthweight.

## Background

Pelvic floor dysfunction (PFD) is a group of disturbances affecting the pelvic floor muscles (PFM) or connective tissues. PFD, including pelvic organ prolapse (POP), urinary and/or anal incontinence, sexual dysfunction, and pelvic pain, affects millions of women around the world. Pregnancy and childbirth are considered high-risk factors for PFD [1].

It is commonly believed that larger infant birthweight is directly related to POP and urinary incontinence. However, the data on this trend are controversial [2–6]. A large cross-sectional study carried out on 21 449 cases in Italy, which involved the largest number of participating individuals to date, demonstrated that macrosomia was not associated with an increased risk of uterine prolapse [5].

Furthermore, the degree of muscle impairment during delivery is positively related to POP. The functional changes in these muscles can be characterized by weak maximum isometric vaginal closure force or maximal voluntary contraction (MVC) of the pelvic floor [7, 8]. In addition, a recent pilot study indicated PFM strength was related to vaginal birth and POP [8]. One longitudinal study involving 1143 participants also found that PFM strength could predict the probability of POP and stress urinary incontinence (UI) within the first two decades after natural labor [9]. Since PFM can be modified by postpartum pelvic floor muscle training, in order to provide adequate early intervention, it is important to understand whether infant birthweight affects the PFM [10]. Therefore, we performed a cohort study to assess the relationship between infant birthweight and vaginal pressure during MVC of the vagina as an indicator of PFM strength in the Chinese population. We hypothesized that larger infant birthweight was an independent risk factor for decreased PFM.

## Methods

### Study design and participants

**Fig. 1:**
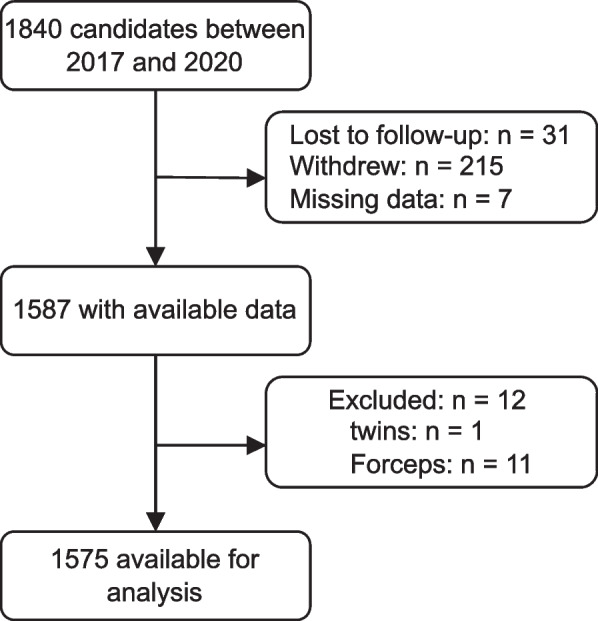
Flowchart of the screening of study participants

We performed a cohort study at Renji Hospital, School of Medicine, Shanghai Jiaotong University to explore the correlation between birthweight and PFM contraction. The study was one component of a program that was designed to explore the influencing factors during pregnancy and delivery for PFD. The purposes of this program are to prevent PFD and improve overall female health. The study was conducted according to the Declaration of Helsinki 1975 and approved by the ethics committee of Renji Hospital, Shanghai Jiao Tong University School of Medicine. Study participants were individuals who delivered vaginally between January 2017 and May 2020 and completed the pelvic floor examination within 5–10 weeks after delivery were recruited. In our hospital, a pelvic floor examination is recommended for all postpartum women. We excluded the participants who delivered twins or had operative vaginal deliveries requiring the use of forceps. Consequently, 1575 individuals were included in this study. A flowchart of the study is represented (Fig. 1).

### Data collection

Demographic information, pre-pregnancy BMI, gestational weight gain (GWG), UI during the third trimester, the circumstances of delivery, and clinical status of newborns were collected from the subjects’ medical histories. Birthweight was divided into 4 groups based on quartiles.

### Outcome variable

The vaginal pressure during MVC was the outcome variable for this research. A neuromuscular stimulation therapeutic instrument, Phenix U4 (Electronic Concept Lignon Innovation, France), which consists of a pressure probe, was used to detect the resting pressure (in centimeters of water) and peak pressure. With the probe in the vagina, participants were instructed to relax the vagina to obtain the resting pressure and to contract the pelvic floor muscles as strongly as possible to obtain the peak pressure. The contraction was repeated 3 times. The difference between the peak pressure and resting pressure represents vaginal pressure during MVC. These pelvic floor electrophysiological parameters were detected by two trained pelvic physiotherapists (Junwen Si, Lu Zhao).

### Covariates

Based on the literature, age [11, 12], pre-pregnancy BMI [12], occupation [4], education [13], stage of labor [11], episiotomy [11], spontaneous perineal tears [14], parity [15], the number of vaginal deliveries [4], gestational age [16], GWG [17], and the occurrence of UI during pregnancy [18] have all been indicated as factors that increase the risk of PFD. In addition, 9.2% of postpartum hemorrhage (PPH) among singleton pregnancies was caused by obstetric trauma, which included perineal laceration, cervical or vaginal trauma, and inversia uteri [19]. Although there are no published studies directly associating PPH with PFD, PPH together with all the above clinical variables was entered into the analysis.

### Statistical analysis

The enumeration data were expressed in number (percentage) and analyzed by Fisher’s exact tests or chi-square tests. The measurement data were tested for normal distribution by Kolmogorov–Smirnov statistic. Data were presented as mean ± standard deviation (SD) when they were normally distributed and analyzed by one-way ANOVA. Data were expressed as median and interquartile ranges when they were abnormally distributed and analyzed by Kruskal–Wallis tests. Multivariable linear regression analyses were used in both crude and adjusted models to explore the relationship between birthweight and vaginal pressure. All the covariates were put into a linear regression model in the basic model to get an initial regression coefficient. Then they were deleted one by one in new models to get new regression coefficients. Those who changed the basic regression coefficients by more than 10% were brought into the study. Thus, the adjusted model was adjusted for age, PPH, and the number of vaginal deliveries. The subgroup analyses were also conducted for all strata, which were significantly different in the univariate analysis. The Statistical Product for Social Science (SPSS, IBM, Armonk, NY, USA) version 23.0 and R 3.4.3 (The R Foundation, Vienna, Austria) were used for all analyses and a p-value below 0.05 was considered statistically significant.

## Results

### Baseline characteristics

Among 1575 subjects from the study, the median duration after delivery was 45 (42–48) days. The mean age was 29.5 ± 3.9 years. The mean birthweight was 3313.6 g ranging from 950 to 4730 g, while the median vaginal pressure was 49.5 cmH_2_0 ranging from 8 to 98 cmH_2_0. Participants with larger infant birthweight had a higher risk of perineal laceration and UI during the third trimester. Infant birthweight was proportional to pre-pregnancy BMI, GWG, postpartum hemorrhage, gestational age, the number of vaginal deliveries, and parity, while inversely proportional to vaginal pressure (Table 1).

**Table 1:** Baseline characteristics of participants by categories of birthweight

Variables	All participants	Birthweight				*P* value
		Quartile 1 ≤ 3045 g	Quartile 2 3046–3310 g	Quartile 3 3311–3570 g	Quartile 4 ≥ 3571 g	
Participants (n)	1575	388	394	397	396	
Vaginal pressure (cmH_2_O)	49.5 (37.5, 66.0)	54.0 (40.5, 73.5)	48.0 (37.5, 66.0)	48.0 (36.0, 63.0)	47.6 (36.0, 61.9)	< 0.001
Age (years)	29.5 ± 3.9	29.6 ± 4.0	29.4 ± 3.8	29.4 ± 4.0	29.7 ± 3.8	0.677
Pre-pregnancy BMI (kg/m^2^)	21.5 (19.8, 23.4)	20.8 (19.4, 22.9)	21.1 (19.7, 22.9)	21.7 (20.0, 23.4)	22.3 (20.3, 24.1)	< 0.001
GWG (kg)	12.0 (10.0, 15.0)	11.5 (9.0, 14.0)	12.0 (9.5, 15.0)	12.0 (10.0, 15.0)	13.0 (10.0, 16.0)	< 0.001
Occupation						0.981
No	303 (30.9)	67 (29.8)	75 (31.4)	79 (31)	82 (31.3)	
Yes	678 (69.1)	158 (70.2)	164 (68.6)	176 (69)	180 (68.7)	
Education						0.577
No and primary	14 (0.9)	4 (1.1)	2 (0.5)	6 (1.6)	2 (0.5)	
Secondary	414 (27.5)	108 (29)	102 (27.2)	95 (24.9)	109 (29)	
Tertiary	1077 (71.6)	261 (70)	271 (72.3)	280 (73.5)	265 (70.5)	
Gestational age (days)	276.0 (271.0, 281.0)	271.5 (265.0, 277.0)	275.0 (271.0, 280.0)	277.0 (274.0, 281.0)	279.0 (274.0, 283.0)	< 0.001
The first stage of labor (min)	240.0 (150.0, 390.0)	240.0 (140.0, 361.2)	240.0 (150.0, 370.0)	240.0 (150.0, 414.0)	270.0 (160.0, 391.2)	0.262
The second stage of labor (min)	25.0 (13.0, 43.0)	22.0 (12.0, 40.2)	26.0 (13.0, 47.0)	25.0 (14.0, 45.0)	25.0 (13.0, 40.0)	0.366
The third stage of labor (min)	5.0 (5.0, 7.0)	5.0 (5.0, 8.0)	5.0 (5.0, 7.8)	5.0 (5.0, 7.0)	5.0 (5.0, 7.0)	0.879
Total stage of labor (min)	283.0 (185.0, 435.0)	270.0 (175.0, 425.0)	271.5 (190.0, 419.0)	288.0 (184.0, 455.0)	305.0 (190.0, 440.0)	0.33
Perineal condition						0.001
Intact	82 (5.2)	18 (4.6)	26 (6.6)	22 (5.5)	16 (4.1)	
Episiotomy	918 (58.3)	247 (63.7)	244 (61.9)	230 (57.9)	197 (49.9)	
I° laceration	514 (32.7)	107 (27.6)	109 (27.7)	133 (33.5)	165 (41.8)	
II° laceration	60 (3.8)	16 (4.1)	15 (3.8)	12 (3)	17 (4.3)	
PPH (ml)	180.0 (130.0, 250.0)	150.0 (120.0, 227.5)	175.0 (130.0, 240.0)	180.0 (130.0, 290.0)	200.0 (140.0, 302.5)	< 0.001
Number of vaginal deliveries						< 0.001
1	988 (62.7)	277 (71.4)	263 (66.8)	243 (61.2)	205 (51.8)	
2	568 (36.1)	108 (27.8)	128 (32.5)	149 (37.5)	183 (46.2)	
3	19 (1.2)	3 (0.8)	3 (0.8)	5 (1.3)	8 (2)	
Parity						< 0.001
1	950 (60.3)	262 (67.5)	255 (64.7)	233 (58.7)	200 (50.5)	
2	597 (37.9)	123 (31.7)	134 (34)	157 (39.5)	183 (46.2)	
3	28 (1.8)	3 (0.8)	5 (1.3)	7 (1.8)	13 (3.3)	
UI during the third trimester	553 (35.1)	120 (30.9)	125 (31.7)	147 (37)	161 (40.8)	0.011

Data presented are mean ± SD, median (Q1, Q3), or N (%)

*Abbreviations*: *BMI* Body mass index, *GWG* Gestational weight gain, *PPH* Postpartum hemorrhage, *UI* Urinary incontinence

### Association of birthweight with vaginal pressure

The beta coefficient (95%) for vaginal pressure was -0.006 (95%CI -0.009 to -0.004, *P* < 0.001) for total subjects. When multivariable linear regression analysis was performed after adjusting for age, postpartum hemorrhage, and the number of vaginal deliveries, the beta coefficient for vaginal pressure was -0.005 (95%CI -0.008 to -0.002, *P* < 0.001) in total subjects, which showed that there was a negative association between birthweight and vaginal pressure. We further categorized subjects into groups according to the clinical diagnostic cut-off value and the quartile of birthweight, respectively. Beta coefficients for vaginal pressure decreased as the level of birthweight increased (*P* = 0.01, *P* for trend < 0.001). In the adjusted model, the association with the birthweight classification group remained significant (*P* = 0.03, *P* for trend < 0.001) (Table 2).

**Table 2:** Association between birthweight and vaginal pressure

Variable	N	Crude mode	Multivariable-adjusted mode
		β (95%CI)	β (95%CI)
Birthweight (g)	1575	-0.006 (-0.009, -0.004)	-0.005 (-0.008, -0.002)
Birthweight (g)	1575		
< 4000	1507	0 (Ref.)	0 (Ref.)
≥ 4000	68	-4.79 (-8.23, -1.36)	-3.29 (-6.75, -0.18)
Birthweight Quartile	1575		
Quartile 1	388	0 (Ref.)	0 (Ref.)
Quartile 2	394	-5.36 (-8.31, -2.41)	-5.04 (-7.98, -2.1)
Quartile 3	397	-6.39 (-9.34, -3.44)	-5.53 (-8.5, -2.57)
Quartile 4	396	-7.52 (-10.47, -4.57)	-6.07 (-9.08, -3.07)
P for trend		< 0.001	< 0.001

Data presented are β and 95% CI. Multivariable-adjusted mode adjusts for age, postpartum hemorrhage, and the number of vaginal deliveries

### Subgroup analyses

**Fig. 2:**
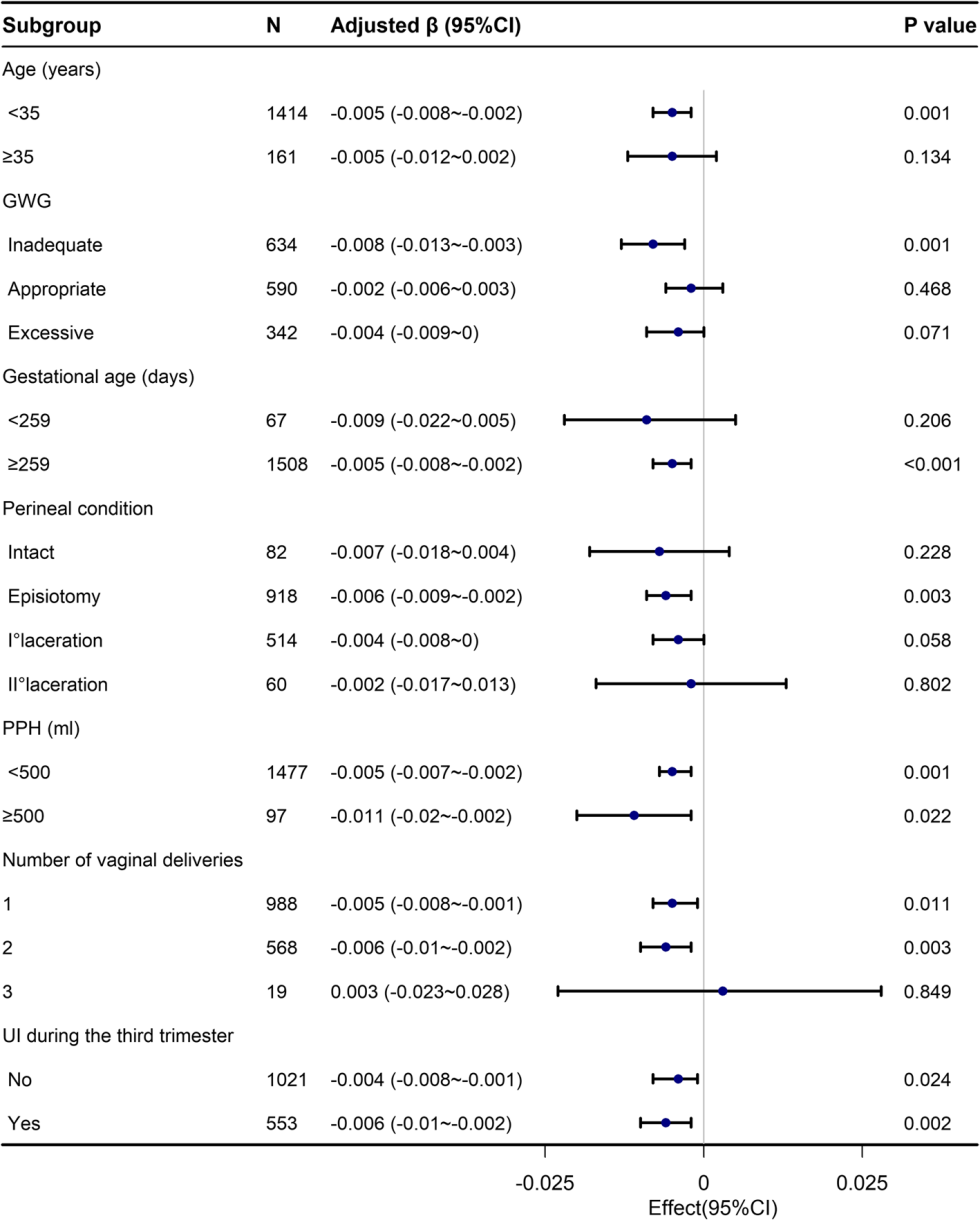
Forest plots of the relationship between vaginal pressure and birthweight by age, GWG, gestational age, perineal condition, PPH, number of vaginal deliveries, and UI during the third trimester. Abbreviations: GWG, gestational weight gain; PPH, postpartum hemorrhage; UI, urinary incontinence

To detect the potential confounders, we performed subgroup analyses based on age, GWG, gestational age, perineal condition, postpartum hemorrhage, number of vaginal deliveries, and UI during the third trimester. The results of subgroup analyses are presented in Fig. 2. Vaginal pressure was associated with infant birthweight among the following participants: aged < 35 years、inadequate GWG、full term pregnancy、histories of one or two vaginal births, or with episiotomies.

## Discussion

We explored the relationship between infant birthweight and PFM strength as measured by vaginal pressure during MVC in the Chinese population. The present cohort study clearly showed that infant birthweight was associated with decreased vaginal pressure, independent of age, PPH, and the number of vaginal deliveries. The results were robust in subgroup analyses.

Measurement of muscle strength, local muscle endurance, muscle force, muscle coordination, synergistic contraction, and neuromuscular control are all methods used to synthetically assess the PFM. There are a variety of methods for the assessment of PFM strength, including palpation, pelvic floor manometry, pelvic floor dynamometry, and electromyography [20]. Since both the interrater and intrarater reliability of manometry and dynamometry were higher than that of palpation and surface electromyography, manometry and dynamometry were considered to be more reliable tools for the measurement of PFM strength [21]. With the popularity of manometry equipment in China, obtaining corresponding data is simple, convenient, and inexpensive. Thus, vaginal pressure during MVC was selected as the independent variable to evaluate and compare PFM strength by considering these factors comprehensively.

Our study revealed that infant birthweight correlated with vaginal pressure during MVC after vaginal birth. The mechanism has not yet been fully elucidated, but we speculate that the following may be contributing factors: the first factor to be considered is the levator ani muscle (LAM) injury. The LAM complex is the main construction of the pelvic floor. At the end of the second stage of labor, LAM is subjected to excessive traction, which may lead to muscle damage. A geometric model has suggested that regions of the pubovisceral, iliococcygeus, and puborectalis muscles reached maximal stretch ratios of 3.26, 2.73, and 2.28, respectively. These values enormously exceeded the allowable maximum stretch ratio of 1.5 tolerated in striated muscle [7]. LAM defect rate was significantly higher in women who delivered larger birthweight infants [22]. The second is the pudendal nerve damage. The pudendal nerve terminal motor latencies (PNTML) examination is used to detect the conduction of the fast motor fibers within the pudendal nerve. The increase of it implies damage to motor fibers. Heavy infants contributed to a significant prolongation of PNTML after childbirth. Moreover, recently, Pipitone et al. found that in addition to muscle tears and defects, LAM injury can be manifested by edema on MRI scans with high spatial resolution. Mean birthweight was 9% greater in women with muscle edema. As a new marker of tissue injury, it helps to detect the LAM injury at a finer level and confirm the effect of newborn birthweight on pelvic floor dysfunction [23].

In most subgroups, the beta coefficient (95%) for vaginal pressure showed a negative relationship between birthweight and vaginal pressure, even if there were no significant differences in some of the subgroups. The interpretation may be due to the limited numbers in these groups reducing statistical power. However, we did not attempt to reclassify these groups since the classification reflects commonly used cut-offs in clinical decision-making. Interestingly, adequate or excessive weight gain attenuated a negative association between birthweight and pelvic floor strength. The conclusions are in accordance with previous studies. Baumann et al. found women with a greater BMI had a lower risk of sphincter laceration postpartum [24]. The possible reason is that the adipose tissue in these women may actually act as a protection to reduce childbirth-related damage to muscle and nerves in the pelvis.

Our cohort has added new information to the existing body of knowledge about birthweight as a high risk for pelvic muscle injury in women after childbirth. Our findings regarding newborn birthweight are in parallel with previous studies about POP and UI [2–4]. These results may provide the extra basis for appropriate fetal weight control during pregnancy. Moreover, PFM strength in women with vaginal delivery is anticipated to increase by 15.7 cm H_2_O after 16 weeks of physical therapy, while it increases slowly over time, by only 3.7 cm H_2_O per five years without training [25, 26]. Thus, women delivering babies with larger birthweight might benefit from earlier pelvic floor rehabilitation. The strengths of this study are the relatively large size and the population-based cohort design. Additionally, with the analysis stratified by age, GWG, gestational age, perineal condition, postpartum hemorrhage, number of vaginal deliveries, and UI during the third trimester, the results were the same as those of the overall analysis, indicating that the results are stable and reliable. The major limitation of our study is its retrospective nature with obvious selection bias. Those who declined to undergo the pelvic floor assessment seems to be less educated and from a lower-income demographic. To reduce the potential bias, we have included in the univariable analysis all the documented variables potentially affecting the PFM strength to screen as many confounders as possible. Another limitation of the study is the lack of examinations on genital prolapse and clinical symptoms records, such as UI and anal incontinence. Therefore, no direct relationship can be established between birthweight and prolapse or incontinence. Also, previous history of operative delivery, which has a significant effect on PFM, was not included as a covariate, since only 11 women experienced a forceps event. In addition, longitudinal studies have shown pelvic floor muscle strength increased by 12.1 cm H_2_O six months compared to six weeks after delivery without intervention. It would be of great interest to conduct the study at six months postpartum. However, there is currently no information available about this. Further studies should be performed to address the issues in this manuscript.

## Conclusions

In conclusion, this study demonstrates that increased infant birthweight was associated with decreased vaginal pressure in women after vaginal delivery and infant birthweight could be considered a risk factor for decreased PFM strength in the population delivering vaginally.

## Data Availability

The datasets used or analyzed during the current study are available from the corresponding author upon reasonable request.

## Abbreviations

PFM: Pelvic floor muscle
PFD: Pelvic floor dysfunction
POP: Pelvic organ prolapse
UI: Urinary incontinence
MVC: Maximal voluntary contraction
SD: Standard deviation
GWG: Gestational weight gain
PPH: Postpartum hemorrhage
LAM: Levator ani muscle
PNTML: Pudendal nerve terminal motor latencies
